# A Possible Association Between COVID-19 Vaccine and Complicated Pleural Effusion: A Case Report

**DOI:** 10.7759/cureus.39133

**Published:** 2023-05-17

**Authors:** Bashar Oudah, Mohammad Abu-Abaa, Noor Al-Ameri, Jonathan Ghazaleh, Vijay Vanam

**Affiliations:** 1 Internal Medicine, Eisenhower Medical Center, Ranch Mirage, USA; 2 Internal Medicine, Capital Health Regional Medical Center, Trenton, USA; 3 Internal Medicine, Eisenhower Medical Center, Rancho Mirage, USA

**Keywords:** pleurisy, vaccine, covid 19, empyema, pleural effusion

## Abstract

As COVID-19 vaccines gain more prevalence, previously unrecognized side effects continue to be reported. We report a case of 78 male with no significant past medical history who was found to have a unilateral pleural effusion with symptoms that started two days after the administration of a COVID-19 vaccine. The initial presumption was bacterial pneumonia with parapneumonic effusion. However, the lack of clinical response prompted surgical intervention, and a diagnosis of empyema was established. No evidence of infectious etiology was found. This case helps to support the previously limited evidence in the recent medical literature that suggests a possible association between COVID-19 vaccines and pleurisy/effusion.

## Introduction

Moderna COVID-19 vaccine is based on a segment of the viral mRNA with a nanoparticle envelope to include host immunity [1]. As the worldwide immunization against COVID-19 infection is gaining more prevalence, uncommon side effects of such vaccines are being reported, including pericarditis, myocarditis, and pleurisy. The association between these occurrences and COVID-19 vaccines cannot be ascertained, but the temporal relationship and the lack of other identifiable causes maintain plausibility.

## Case presentation

A 78 years old male with the unremarkable past medical history presented to the emergency department (ED) during the summer with a one-week history of pleuritic chest pain, cough, and shortness of breath. He could not recall any preceding event or symptoms except that these symptoms started two days after receiving the Moderna Covid-19 booster. In ED, he was found to be hypoxic with an oxygen saturation of 85% on room air, tachycardic with a heart rate of 118 beats per minute, and tachypneic with a respiratory rate of 25 cycles per minute. Hypoxia was correctable with supplementary oxygen therapy at 2 liters. However, he was afebrile with stable blood pressure. Physical exam was significant for diminished breath sounds over the left lower third of the lung field with decreased vocal fremitus and dull percussion note. Otherwise, the physical exam was unremarkable. 

**Figure 1:**
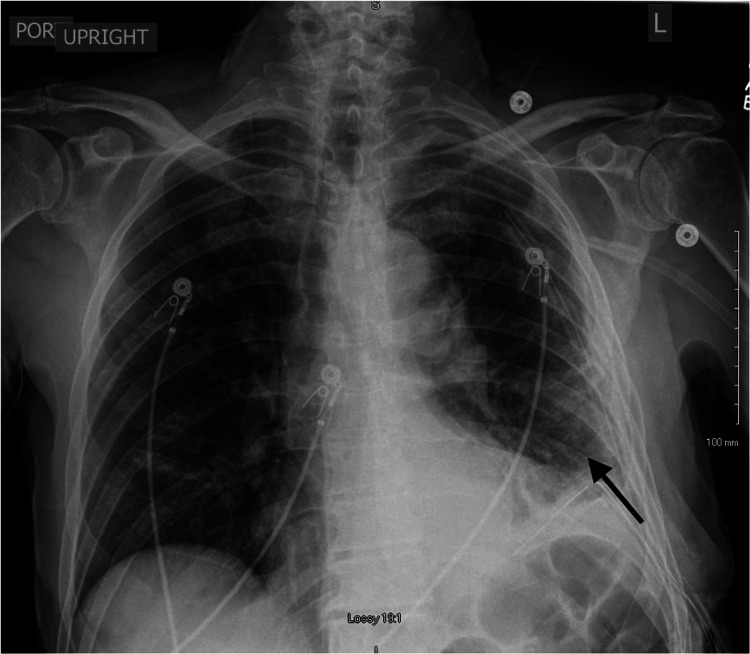
Initial Chest X-Ray An upright posterior-anterior chest x-ray showing left-sided lower lung opacity with obliterated costophrenic angle suggestive of pleural effusion (arrow).

**Figure 2:**
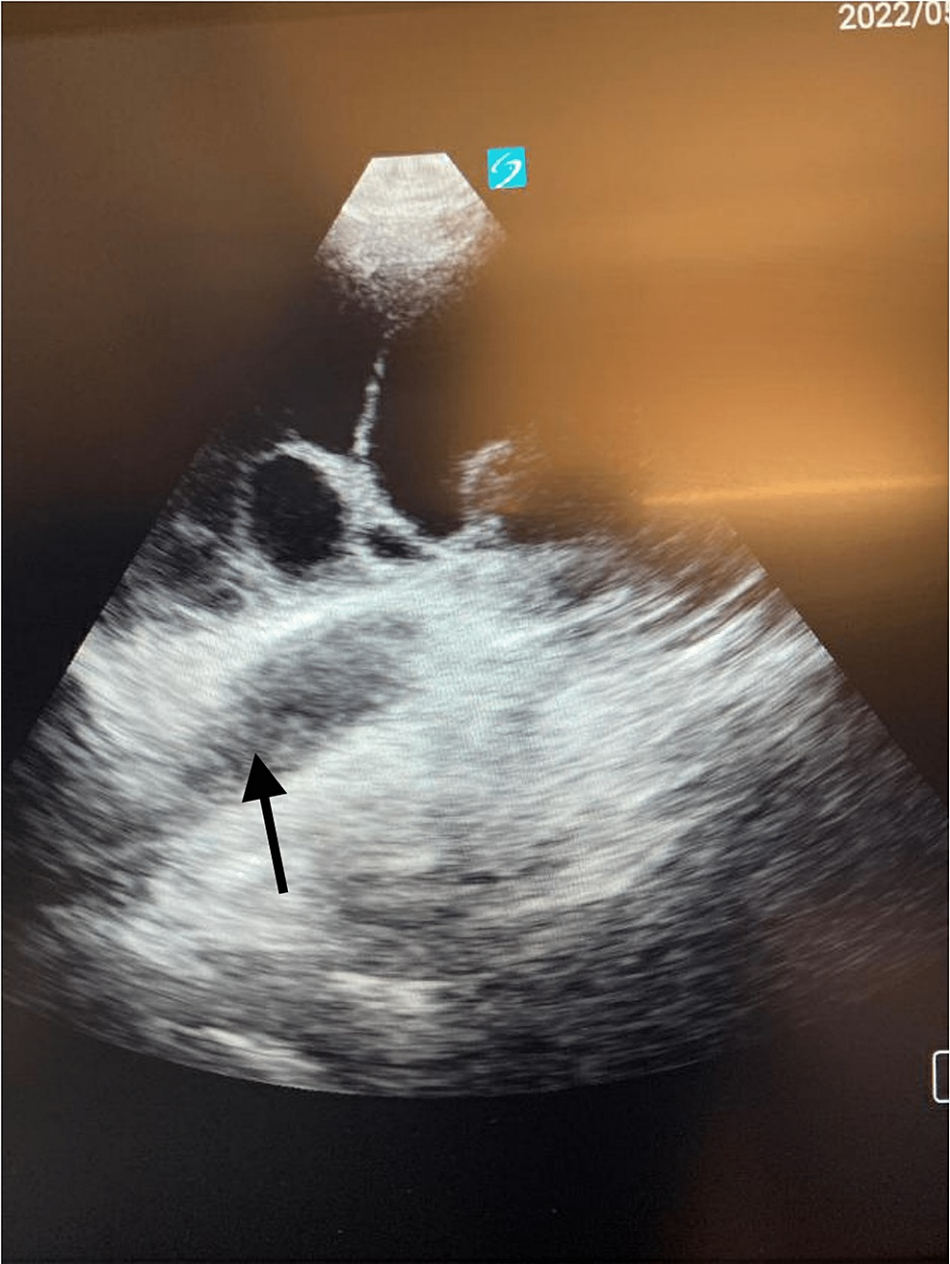
Transthoracic Echocardiography A transthoracic echocardiography showing fluid collection adjacent to the heart suggests pleural effusion (arrow).

**Figure 3:**
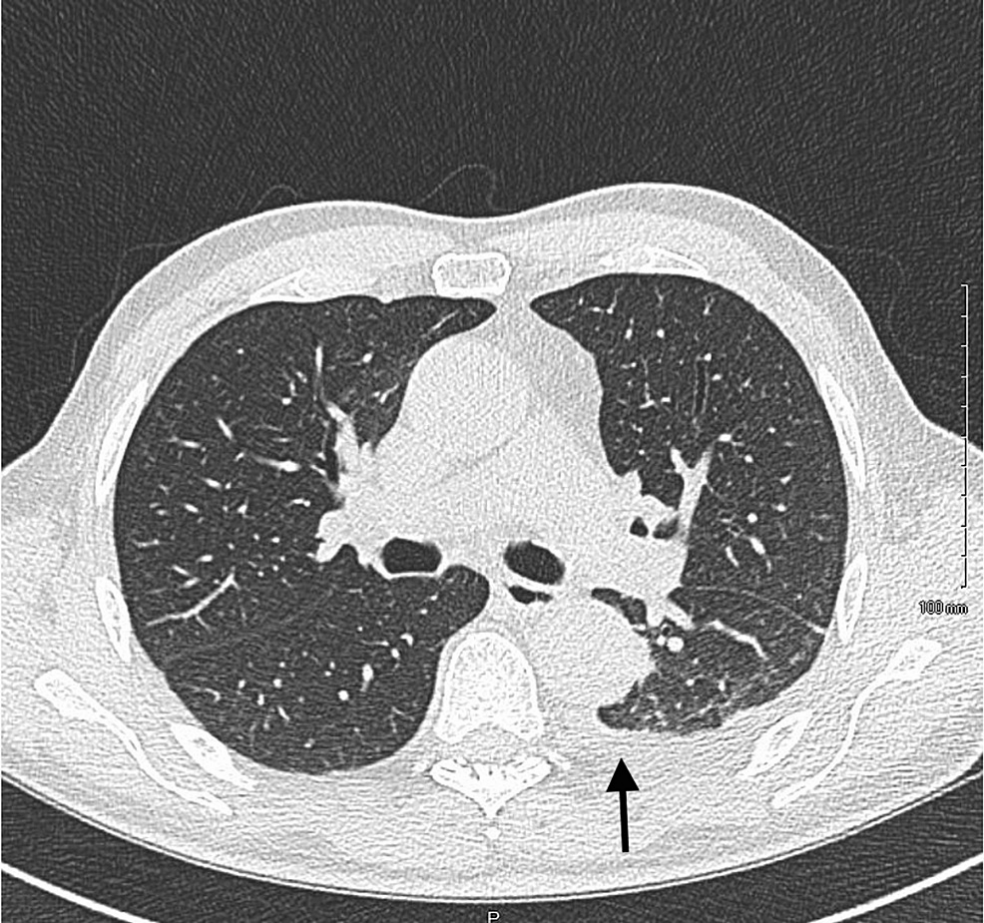
Chest Computed Tomography (CT) Scan A chest computed tomography (CT) showing a left-sided posterior fluid collection (arrow).

**Figure 4:**
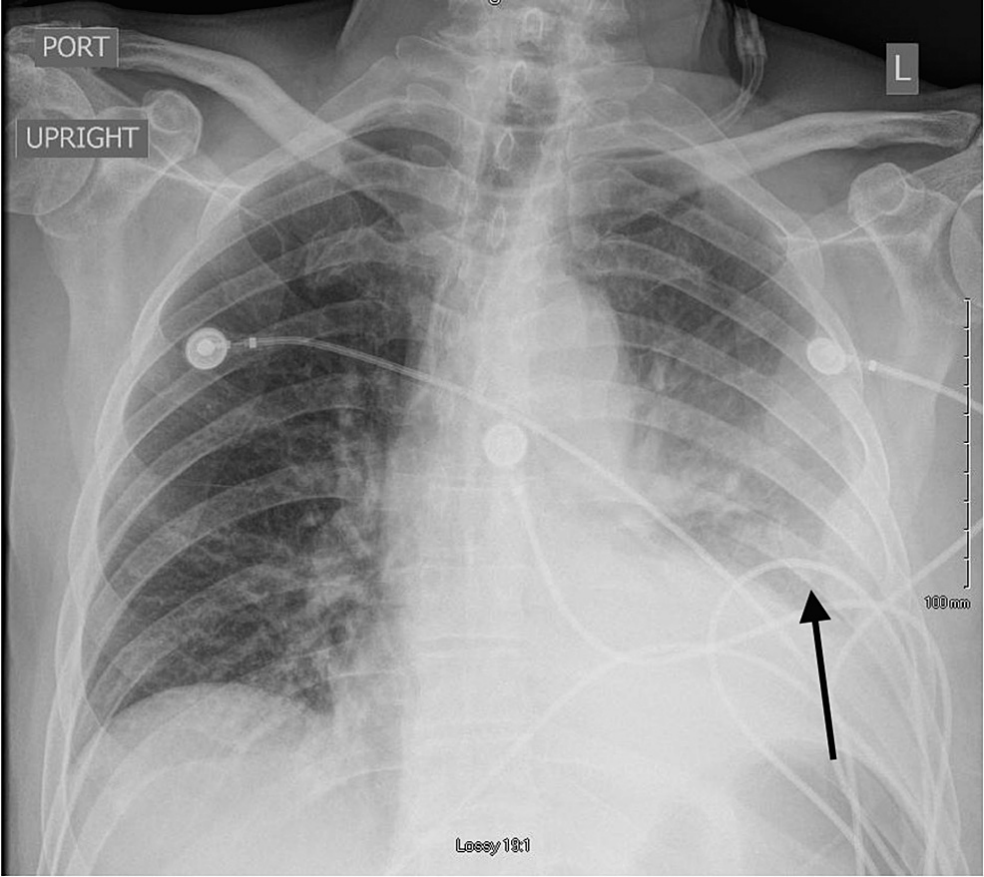
Follow-up Chest X-Ray A follow-up chest x-ray showing the progression of the left-sided opacity (arrow).

Basic labs showed leukocytosis of 26x10^3^ cells/mcl, hyponatremia of 130 mmol/L, hypokalemia of 2.9 mmol/L with evidence of acute kidney injury with elevated serum creatinine level to 1.4 mg/dl from a normal baseline. Chest x-ray showed large left lower lobe opacity with obliteration of the costophrenic angle suggestive of pleural effusion (Figure 1). Blood cultures, as well as a sputum culture, were obtained. He was admitted to the hospital for a presumed diagnosis of community-acquired pneumonia and started on Ceftriaxone and Azithromycin. Electrocardiography (EKG) was unremarkable. A transthoracic echocardiogram was unremarkable for cardiac findings. A bedside ultrasound (US) showed pleural effusion on the left side with pulmonary parenchymal echogenicity (Figure 2). This was also demonstrated by computed tomography of the chest, which showed a left-sided pleural effusion with no visible pulmonary parenchymal infiltrate/consolidation (Figure 3). While hospitalized, a follow-up chest x-ray revealed worsening pleural effusion despite appropriate treatment (Figure 4). Diagnostic thoracentesis was pursued and yielded 200 ml of serosanguineous fluid, and antibiotics were broadened to Piperacillin-tazobactam. Pleural fluid analysis showed exudative fluid with elevated protein 4.6 mg/dl, elevated LDH of 3.728 IU/L, very low glucose of less than 10 mg/dl, and very high WBC count of 17584 cell/ml, see (Table 1, 2). Gram stain and pleural fluid cultures, including bacterial, fungal, and acid-fast bacilli cultures, were unyielding. Also, cytology was negative. Blood cultures remained negative, and the respiratory viral panel was also unyielding. A bedside ultrasound (US) revealed a complex effusion with multiple thick loculations.

**Table 1:** Lab Findings on Admission and Follow-Up WBC: white blood cells, Hb: hemoglobin.

Variable	On Admission	2 months after	Reference Range
WBC	26	6.4	4-10^3^ cells/mcl
Hb	12.6	11.2	13-16 g/dl
Platelet count	214	345	150-400x10^3^ cells/mcl
Sodium	130	135	135-145 mmol/L
Potassium	2.9	4.5	3.5-5 mmol/L
Creatinine	1.4	0.9	Less than 1 mg/dl
Albumin	2.6	4.2	3.5-5 g/dl
Total protein	5.8	7.3	4-6.5 g/L

**Table 2:** Pleural Fluid Analysis RBC: red blood cells, WBC: white blood cells, pH: potential of hydrogen.

Body fluid appearance	Cloudy
Body fluid color	yellow
eosinophils	0
basophils	0
lymphocytes	1
monocytes/macrophages	0
RBC count	2000
WBC count	17584
Cholesterol, body fluid	93
Glucose, fluid	<10
pH, body fluid	7.1
Triglycerides, body fluid	53
neutrophils, body fluid	99

**Figure 5:**
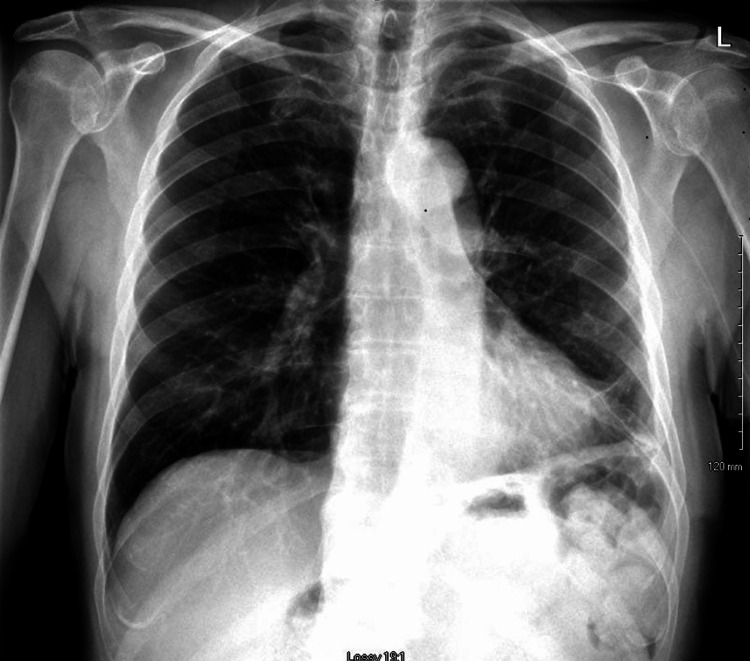
Post-Surgical Chest X-Ray A post-VATS chest X-ray showed the resolution of left-sided pleural effusion.

Given the lack of response with broad-spectrum antibiotics, the lack of evidence of infection by cultures, and the multiloculated nature of pleural effusion, the decision was to proceed with video-assisted thoracoscopic decortication (VATS). This has yielded 1100 ml of serosanguineous exudate. Chest tube output was and allowed the drainage of a further 250 ml of serosanguinous fluid in the first 12 hours postoperatively. However, afterward, drainage was minimal. The tube was removed three days afterward. Repeat chest x-ray after removal of chest tube revealed resolution of the effusion (Figure 5). No evidence of recurrence of pleural effusion was noted during follow-up a few months later.

## Discussion

Pleural/pulmonary inflammation secondary to COVID-19 vaccines has rarely been reported in the literature, and hence there is no exact epidemiological data [2-4]. Pleural effusion has been reported as an isolated finding [5] and is associated with pericarditis/pericardial effusion [4,6]. The interval between the administration of the COVID-19 vaccine and the occurrence of pleurisy/pleural effusion ranged between 1-2 weeks [2-4] but was also reported as early as 3 days after vaccine administration [6]. Pleural effusion has also been reported in association with polyarthritis after the COVID-19 vaccine [7]. On the other hand, the incidence of pleural effusion in those with COVID-19 disease is low at only 5% [8]. Pleural effusion in this setting is perceived as a marker of poor prognosis and is likely secondary to systemic inflammatory syndrome and cytokine storm, given the noted decrease in lymphocytes and increase in platelets, CRP, and LDH in pleural effusion [9].

The etiology of pleural effusion varies from idiopathic, viral, bacterial, and tuberculous infection to autoimmune disease and malignancy. Diagnosis of complicated pleural effusion, in this case, was based on a low pleural fluid glucose level of less than 60 mg/dl along with pleural fluid PH less than 7.20 with no drained pus and persistently negative cultures and gram stain [10]. The suggestion of the association between complicated parapneumonic pleural effusion and the COVID-19 vaccine is plausible, given the temporal relationship and unyielding extensive infectious work, including unyielding blood cultures, sputum cultures, pleural fluid cultures, and viral panels. No evidence of non-infectious etiologies was also seen, and no pulmonary infiltrate/consolidation was appreciated on imaging.

Post-marketing pharmacovigilance remains vital to establishing an accurate safety profile of COVID-19 vaccines [11]. The currently available evidence of a possible association between COVID-19 vaccines and pleural/pericardial effusion is only based on a few cases reports [11]. No report of pleural effusion is listed among the potential complications of the Moderna vaccine in the Summary of Product Characteristics [12]. To our knowledge, only one case of pleuro-pericardial effusion secondary to the Moderna COVID-19 vaccine was reported previously in Spain [13]. However, unlike our case, pleural effusion, in that case, was part of a multisystemic inflammatory syndrome. Recently, Two cases were reported describing polyserositis involving pericardial and pleural effusion [11,14]. A hemorrhagic variant of pleural effusion was also reported [4].

The exact pathological mechanism responsible for this complication remains poorly understood. However, the current most acceptable theory of mRNA vaccine-induced pleural effusion is molecular mimicry [15]. The spike proteins of the SARS-CoV-2 virus have homological similarities to many human proteins, including the alveolar surfactant protein, triggering immunological reactions against these proteins and hence pulmonary/pleural inflammation [16]. Most cases reported previously in the literature involve pleurisy and pericarditis [15]. Manifestations of IgG-4-related disease, including pleural effusion, after the COVID-19 vaccine, also have been reported [2,3]. This further supports the molecular mimicry theory as a plausible pleural/pulmonary inflammation mechanism. It is noteworthy that a recent in-depth evaluation of a COVID-19 vaccine-related-myocarditis noted that the antibody response was not different from vaccinated controls. However, an increase in a specific subset of natural killer cells of uncertain significance was noted [17].

## Conclusions

The causal relationship between COVID-19 vaccines and pleurisy/pleural effusion cannot be ascertained based on the currently available limited data, given the rarity of such occurrence. Clinicians should be aware of the COVID-19 vaccine as a possible underlying cause in patients presenting with pleural effusion that otherwise remains unexplained, that causality cannot be established in this case, and that COVID-19 vaccines remain the cornerstone of disease prevention for COVID-19. In this context, infectious etiologies cannot be ruled out completely. Clinicians should remain vigilant of such possibilities, as early establishment of the diagnosis and treatment can improve the outcome. COVID-19 vaccines remain highly recommended to the public.
